# Possible Mechanisms Underlying Neurological Post-COVID Symptoms and Neurofeedback as a Potential Therapy

**DOI:** 10.3389/fnhum.2022.837972

**Published:** 2022-03-31

**Authors:** Mária Orendáčová, Eugen Kvašňák

**Affiliations:** ^1^Department of Medical Biophysics and Medical Informatics, Third Faculty of Medicine, Charles University in Prague, Prague, Czechia; ^2^Department of Medical Biophysics and Medical Informatics, Third Faculty of Medicine, Charles University in Prague, Prague, Czechia

**Keywords:** post-COVID symptoms, neurological complications, mechanisms, therapy, dynamical system theory, neurofeedback

## Abstract

Theoretical considerations related to neurological post-COVID complications have become a serious issue in the COVID pandemic. We propose 3 theoretical hypotheses related to neurological post-COVID complications. First, pathophysiological processes responsible for long-term neurological complications caused by COVID-19 might have 2 phases: (1) Phase of acute Sars-CoV-2 infection linked with the pathogenesis responsible for the onset of COVID-19-related neurological complications and (2) the phase of post-acute Sars-CoV-2 infection linked with the pathogenesis responsible for long-lasting persistence of post-COVID neurological problems and/or exacerbation of another neurological pathologies. Second, post-COVID symptoms can be described and investigated from the perspective of dynamical system theory exploiting its fundamental concepts such as system parameters, attractors and criticality. Thirdly, neurofeedback may represent a promising therapy for neurological post-COVID complications. Based on the current knowledge related to neurofeedback and what is already known about neurological complications linked to acute COVID-19 and post-acute COVID-19 conditions, we propose that neurofeedback modalities, such as functional magnetic resonance-based neurofeedback, quantitative EEG-based neurofeedback, Othmer’s method of rewarding individual optimal EEG frequency and heart rate variability-based biofeedback, represent a potential therapy for improvement of post-COVID symptoms.

## Introduction

Neurological post-COVID complications include long-term presence of the symptoms such as headache, insomnia, depression, anxiety, dizziness, seizures and mood swings (Asadi-Pooya and Simani, 2020; Caronna et al., 2021; Fernández-De-las-Peñas et al., 2021c; Townsend et al., 2021). They may originate from neural or extra-neural COVID-19-related pathology (Libby and Lüscher, 2020). The prevalence of post-COVID complications ranges from 8 to 47.5% (Fernández-De-las-Peñas et al., 2021d; Kayaaslan et al., 2021; Pilotto et al., 2021) thereby making it likely to be a public health threat and a formidable challenge for a health care system. Neurological complications associated with COVID-19 may exacerbate either during the acute Sars-CoV-2 infection or during its post-acute phase (Collantes et al., 2021; Delorme et al., 2021; Fernández-De-las-Peñas et al., 2021a,e). In this hypothetical article, we propose that there are several pathological processes responsible for exacerbation and persistence of neurological disturbances in acute and post-acute phase of Sars-CoV-2 infection. We postulate that these mechanisms can take place during acute COVID-19, during post-acute COVID-19 or during the both phases. In the second part of this work, we will try to discuss post-COVID complications from the perspective of dynamical system theory using its underlying concepts such as attractors, system parameters and criticality of system behavior. In the final part of this paper, we will discuss the potential beneficial effects of biofeedback therapy on post-COVID neurological complications.

## General Characteristics of COVID-19-Related Neurological Symptoms

COVID-19-related complications include long-term disturbances occurring in nervous, cardio-respiratory, immune, endocrine and gastro-intestinal body systems (Ellul et al., 2020; Dennis et al., 2021; Selvaraj et al., 2021). Some post-COVID symptoms seem to be attributed to the isolated dysfunction of a single body system whereas other symptoms may stem from COVID-19-related dysfunction of multiple body systems. For example, symptoms such as anosmia and ageusia are likely to be caused by isolated dysfunction of nervous system. On the other hand, COVID-19-related fatigue, which is defined as reduction of physical and mental performance due to COVID-19, can be caused by dysfunction of both neural and extra-neural systems (Rudroff et al., 2020; Bilgin et al., 2021; Morgul et al., 2021; Workman et al., 2021). Long-term post-COVID complications occur in COVID-19 survivors regardless their age (Morand et al., 2021) and sex (Blitshteyn and Whitelaw, 2021; Delorme et al., 2021; Fernández-De-las-Peñas et al., 2021b). Female sex was consistently found to be associated with the higher risk of increased probability of exacerbation of long-lasting post-COVID disturbances (Mahmud et al., 2021; Stavem et al., 2021; Thye et al., 2022). The longer recovery period for some post-COVID disturbances such as anosmia and ageusia was observed in women (Meini et al., 2020). Interestingly, some post-COVID complications seem to occur more frequently in men and vice versa, some symptoms are more frequent in women (Meini et al., 2020; Huang et al., 2021; Xiong et al., 2021). For instance, fatigue and myalgia are more common in women (Huang et al., 2021; Stavem et al., 2021; Xiong et al., 2021). On the contrary, anosmia and ageusia were documented to occur more frequently in men (Meini et al., 2020). To the best of our knowledge, it is currently unknown what underlying causes are responsible for these sex differences. Prolonged time for clinical improvement of COVID-19 symptoms and long duration of acute Sars-CoV-2 infection measured by duration of positive RT-PCR test were found to represent risk factors for increased probability of occurrence of long-term post-COVID-19 complications (Mahmud et al., 2021). In addition, some acute COVID-19 symptoms, such as dyspnea (Stavem et al., 2021), chest pain (Walsh-Messinger et al., 2021), fatigue (Mahmud et al., 2021), fever (Mahmud et al., 2021; Walsh-Messinger et al., 2021), headaches (Walsh-Messinger et al., 2021) and olfactory impairment (Walsh-Messinger et al., 2021), were associated with the higher probability of exacerbation of post-COVID complications. In relation to the link between severity of initial acute COVID-19 symptoms and the higher risk of occurrence of post-COVID problems, some studies found the correlation between these two entities (Stavem et al., 2021) whereas other studies did not (Townsend et al., 2020).

In this theoretical work, we will focus on neurological manifestations of COVID-19. Neurological disturbances linked with acute and post-acute period of COVID-19 include disturbances such as dizziness, headaches, epileptic seizures, paresthesia, fatigue, anxiety, depression, sleep disturbances, cognitive dysfunctions and others (Fernández-De-las-Peñas et al., 2021c; Sun et al., 2021). Neurological COVID-19-related disturbances may originate from dysfunctions related to central nervous system (CNS), peripheral nervous system (PNS), autonomous nervous system (ANS), but they may also stem from dysfunctions of extraneural sorgans (Ayat et al., 2021). Since post-COVID-19- symptoms may overlap with the acute COVID-19 symptoms (Mahmud et al., 2021), we postulate that there can be some common mechanism responsible for exacerbation and occurrence of acute and post-acute COVID-19-related disturbances. In the following chapter, we will discuss potential similarities and differences between the pathological processes which may be possibly responsible for neurological complications occurring in acute and post-acute period of COVID-19.

## Mechanisms Responsible for the Exacerbation and Maintenance of COVID-19-Related Neurological Complications

Acute COVID-19 infection may be accompanied with the neurological complications such as headaches, dizziness, seizures, sleep disturbances, anxiety, depression, alterations of taste and smell etc. (Mao et al., 2020; Najjar et al., 2020; Paterson et al., 2020; Collantes et al., 2021). However, these neurological conditions sometimes persist for a long-term period after acute infection, or alternatively, they may exacerbate and develop with some latency for a longer period following acute infection (Delorme et al., 2021; Fernández-De-las-Peñas et al., 2021a). If there is no other objective explanation for the etiology of neurological complications following COVID-19 and at the same time if they occur/persist for more than 3 months after acute COVID-19 infection, then there are attributed to be direct or indirect result of COVID-19 infection and are termed as post-COVID complications (Fernández-De-las-Peñas et al., 2021e).

There are probably several pathological processes responsible for COVID-19-related neurological problems: direct and indirect damage to CNS associated with Sars-CoV-2, long-term recovery of damaged neural tissue, dysfunction of extraneural tissues, psychological factors and mutual co-occurrence and interference between the multiple COVID-19-related symptoms. We believe that these pathogenetic processes may take place in two phases, namely, during the phase of acute Sars-CoV-2 infection and during its post-acute phase. In this chapter, we will discuss possible differences and similarities between these two phases of the aforementioned pathogenetic processes.

### COVID-Related Damage to Neural Tissue

The evidence of the link between COVID-19 and neuronal injury comes from studies which detected elevated serum levels of neurofilament light chain protein, which is the marker of neuronal injury, in mild-to-moderate and moderate-to-severe cases of COVID-19 (Ameres et al., 2020; Kanberg et al., 2020). In relation to COVID-19, damage to neural tissue, responsible for the exacerbation of various neurological disturbances, can manifest as a result of direct and indirect interaction of Sars-CoV-2 with the CNS of the host (Ellul et al., 2020; Mayi et al., 2021; Meinhardt et al., 2021).

#### Direct Damage to Brain Caused by Sars-Cov-2

In acute COVID-19, as a direct viral entry to CNS, pathways through angiotensin 2 (ACE2) and neuropilin-1 in olfactory epithelium has been strongly considered (Ellul et al., 2020; Lukiw et al., 2020; Meinhardt et al., 2021). This can be supported by findings of the study done by Bryche et al. (2020), in which damaged olfactory epithelium has been associated with Sars-Cov-2 infection (Bryche et al., 2020). Also, hematogenic route has been proposed (Kumar et al., 2020; Bodnar et al., 2021). Several autopsy studies done on the victims of COVID-19 patients found evidences of neurotropism of Sars-CoV-2 as the viral RNA has been detected in a various brain regions such olfactory system, brainstem, cerebellum (Meinhardt et al., 2021) and frontal lobes (Gagliardi et al., 2021). Apart from neurons, Sars-CoV-2 presence was detected in astrocytes as well (Crunfli et al., 2021). It may be important to mention that it is possible that dynamics and replication of Sars-CoV-2 may differ with regard to the particular brain cells. In Crunfli et al. (2021) study, both neurons and astrocytes have been infected but the vast majority of infected cells were represented by astrocytes. Viability of the astrocytes was showed to be reduced (Crunfli et al., 2021). Based on these findings and different roles of neurons and glias, it might be interesting to study whether and how clinical representations of COVID-19-related symptoms differ between those patients with prevalent Sars-CoV-2 infection in neurons and in those in whom Sars-CoV-2 predominantly infected astrocytes. The exact mechanisms of interaction between Sars-CoV-2 and neural host cells and their pathological consequences remain elusive so far. There are some implications that Sars-CoV-2 is capable of alterations of gene expressions since it was found that Sars-CoV-2 presence in frontal lobes is associated with down-regulation of genes connected to hypoxia and up-regulations of hemoglobin genes (Gagliardi et al., 2021).

Not much is known about the long-term presence of Sars-CoV-2 in brain in post-acute period of COVID-19. Positive Sars-CoV-2 presence was documented in cytological samples of olfactory mucosa in the participants with and without anosmia approximately 6 months after the initial Sars-CoV-2 infection (de Melo et al., 2021). Viral load was found to be significantly higher in post-COVID-19 participants with long-lasting or relapsing anosmia than in those with no anosmia (de Melo et al., 2021) which suggests positive association between long-lasting viral presence in brain and neurologic symptoms. Damage to the olfactory neuro-epithel was indirectly proven *via* positive detection of increased caspase-3 signal indicating cell death caused by apoptosis (de Melo et al., 2021). Taking into consideration fact that nasopharyngeal RT-PCR was negative in the participants, it is possible that acute and post-acute phases of Sars-CoV-2 infection, accompanied by the presence of Sars-CoV-2 in the brain, differ with regard to the dynamics of distribution of the virus in the various parts of the organism since acute and sub-acute period of Sars-Cov-2 infection is frequently linked with positive nasopharyngeal positive RT-PCR test (Wang et al., 2020; Zhou et al., 2021).

#### Damage to Brain Tissue Caused Indirectly by Sars-CoV-2

Neurological symptoms connected to the acute period of Sars-CoV-2 infection can manifest even without the presence of Sars-CoV-2 in the brain tissue (Ellul et al., 2020). One of the possible explanations for this phenomenon may consist in damage to brain tissue caused by indirect effects of Sars-CoV-2 infection. Indirect effect of Sars-CoV-2 infection may include maladaptive-immune response to the infection (Hussman, 2021),thromboembolism (Wichmann et al., 2020), endothelial dysfunction (Libby and Lüscher, 2020), hypoxia and neurotoxic metabolities released from extraneural organs affected by COVID-19 (Wu et al., 2020. All these factors can cause damage to blood-brain barrier, infiltrate to CNS and cause further damage to brain tissues (Chaparro-Huerta et al., 2005; Takagishi et al., 2010; Slevin et al., 2020; Bobermin and Quincozes-Santos, 2021). In acute COVID-19, neural damage is likely to be caused primarily due to direct or indirect interaction of Sars-Cov-2 with the host organism. This may lead to various neurological consequences. Documented post-mortem brain lesions of COVID-19 non-survivors (Coolen et al., 2020) and neural atrophy associated with the presence of neurological complications were documented in acute COVID-19 (Chiu et al., 2021). This may support the hypothesis of occurrence of neurological problems due to damage to brain tissue caused by Sars-CoV-2 infection.

Apart from neurological complications and brain lesions linked with acute COVID-19, neurological complications accompanied by atrophy of a various brain regions were already documented in post-acute period of COVID-19 (Carroll et al., 2020; Douaud et al., 2021). We believe that one of the possible mechanisms responsible for this phenomenon may include secondary damage to neural tissue. Secondary damage to neural tissues can potentially come into play as a consequence of mechanisms responsible for long-term post-COVID complications. There are probably multiple possible ways of how such secondary damage to neural tissue may arise. For instance, breathing problems such as dyspnea, pulmonary and cardiological pathologies, which occur in post-COVID condition (Goërtz et al., 2020; Zarei et al., 2021) may lead to insufficient distribution of oxygen and/or blood to brain causing hypoxia-related damage to neural tissue. Another possible mechanism may consist in excitotoxic effects of some pro-inflammatory cytokines (Takagishi et al., 2010; Slevin et al., 2020) which may initiate apoptosis in some neural tissues (Chaparro-Huerta et al., 2005). Some post-COVID-19 neurological complications were found to be associated with elevated levels of excitotoxic pro-inflammatory cytokines (Lorkiewicz and Waszkiewicz, 2021; Sun et al., 2021). It is therefore possible that elevated concentrations of these pro-inflammatory cytokines may cause secondary damage to neural tissue which can lead to increasing severity and long-term persistence of the particular existing neurological problems and/or to exacerbations of new neurological pathologies.

We believe that neuroimaging studies done before, during and after acute period of COVID-19 might help to distinguish between primary damage to brain tissue associated to acute COVID-19 and secondary brain damage associated with post-COVID period. Although, both processes can probably overlap in some points, predominance of brain injury in acute COVID-19 might point toward pathogenesis linked with primary damage associated with Sars-CoV-2 infection. On the other hand, the predominance of occurrence of brain injury in Sars-CoV-2 negative COVID-19 survivors in post-acute period might indicate predominance of secondary brain damage linked with post-infectious condition.

### Long-Term Recovery of Damaged Neural Tissue

Long-term recovery of direct or indirect damage of neural tissue, associated with Sars-CoV-2 infection was suggested to be one of the possible causes responsible for the persistence of long-term COVID-19 neurological complications (Yong, 2021). The large time window during which the slow recovery of damaged neural tissues takes place, may give rise to a wide spectrum of a various possible pathophysiological processes which may occur and contribute to long-term and complex post-COVID-19 neurological complications. Furthermore, compensatory brain plasticity which may occur in the period of recovery of neural tissues, may sometimes turn into maladaptive plastic processes (Takeuchi and Izumi, 2012). For instance, hypertrophy of the affected brain area (Shaikh et al., 2010) and compensatory neurogenesis (Lu et al., 2020) were proposed to the possible causes responsible for the volume increase of the selected brain regions in post-COVID state (Lu et al., 2020). Due to possible emergence of maladaptive plasticity in the processes of brain recovery and its long-lasting period, compensatory plasticity might represent a risk factor for long-lasting persistence of post-COVID-19 symptoms and/or for exacerbation of secondary neurological complications.

In the connection to the possible link between COVID-19 and occurrence of long-term recovery of damaged neural tissues and compensatory maladaptive plasticity, we postulate that neurological problems originating from the processes linked with long-term neural recovery are more likely to manifest in sub-acute and/or post-acute phase of Sars-CoV-2 infection than in the early phases of the acute infection.

### Immune-Mediated Adaptation of Central Nervous System

Neurological complications linked with acute COVID-19 may not be always accompanied by damage to a neural tissue (Solomon et al., 2020). One of the possible explaining mechanisms may consist in adaptation of CNS to ongoing inflammatory processes. In order to differentiate between immune-mediated adaptation of CNS to inflammatory processes and damage to neural tissues caused by excitotoxic influence of maladaptive immune response to Sars-CoV-2, which was mentioned in the previous chapter, we feel that definitions of both are necessary to be mentioned in this section. Damage to neural tissues, caused by excitotoxic influence of maladaptive immune response to Sars-CoV-2, is defined as a pathological process leading to the death of neurons. On the other hand, immune-mediated adaptation of CNS to inflammatory processes is defined as quantitative or qualitative change of activation patterns of neurons caused by their interaction with pro-inflammatory cytokines. Even though these two processes are probably not mutually exclusive, in the following section we would focus solely on our perspective of possible immune-mediated adaptation of CNS to acute and post-acute COVID-19.

During inflammatory condition, such as viral or bacterial infection, there comes to a rapid increase of pro-inflammatory markers such as interleukin 4 (il-4), interleukin 6 (il-6), tumor necrosis factor (TNF) and C reactive protein (CRP) (Slaats et al., 2016). Elevated levels of these cytokines are frequently present in acute COVID-19 (Effenberger et al., 2021). The role of these cytokines seems to consist not only in a successful mobilization of immune system to combat the pathogen, but it also seems to consist in a modulation of CNS functioning with regard to ongoing infectious processes (Felger, 2017). Pro-inflammatory cytokines were found to be capable of affecting regions such as amygdala, insula, cingulate gyrus, prefrontal cortex (PFC) (Felger, 2017). They can propagate into CNS by the following ways: through leak regions in blood brain barrier (BBB), by inducing of activation of cytokine uptake mechanisms in BBB and by activation of afferents of vagal nerve which are capable to relay cytokine signals to relevant brain structures (Felger, 2017). Increased levels of inflammatory markers were found to alter a variety of brain regions, for instance, in chronic fatigue syndrome, elevated concentrations of pro-inflammatory cytokines are associated with reduced activity in reward-related neuronal circuitries (Capuron et al., 2012). On the other hand, elevated levels of il-6 and TNF were coupled with increased activity in amygdala associated with the feelings of a social disconnection and socially threatening images (Inagaki et al., 2012). From the evolutionary point of view, immune-mediated inhibition of reward systems and increased activation of brain structures responsible for a greater awareness of a potential threats, were proposed to serve as adaptive behavioral adjustment of CNS activity to ongoing infection (Felger, 2017). Its main purpose is thought to consist in decreasing motivation of exploratory behavior and mobility that would prevent from a successful recovery from ongoing infections (Felger, 2017).

In relation to post-acute COVID-19 period, this kind of immune-mediated adaptations of CNS might probably occur as well, for instance, due to the presence of a long-term persistence of SARS-CoV-2 in brain or its presence in some other tissues. Immune-mediated adaptation of CNS to long-term elevations of pro-inflammatory cytokines may possibly also come into play as a result of dysautonomia which can occur in COVID-19 survivors (Barizien et al., 2021; Blitshteyn and Whitelaw, 2021; Goodman et al., 2021). Elevated levels of some pro-inflammatory markers may lead to decreased activity of reward neural circuitry and increased activity in the parts of amygdala responsible for fear-related responses (Felger, 2017). Such alterations in CNS functioning might likely lead to conditions such as permanent anxiety, mood disturbances, insomnia and depression, which are quite a frequent post-COVID problems (Fernández-De-las-Peñas et al., 2021c; Lorkiewicz and Waszkiewicz, 2021; Townsend et al., 2021). This hypothesis might be at least partially supported by the fact that brain structures, which are responsive to cytokine signaling such as insula, amygdala and gyrus cingulate (Felger, 2017) were repeatedly found to exhibit various abnormalities in post-COVID-19 condition (Lu et al., 2020; Guedj et al., 2021a; Morand et al., 2021; Sollini et al., 2021). Also, elevated levels of pro-inflammatory cytokines were found to accompany neuropsychological disturbances such as depression and anxiety (Young et al., 2014; Tang et al., 2018) thereby speaking in favor of the existence of the potential link between post-COVID neurological and psychiatric disturbances and immune-mediated adaption of CNS to COVID-related inflammatory processes. This may be at least partly supported by positive correlation between the level of pro-inflammatory cytokines and severity of post-COVID depression (Lorkiewicz and Waszkiewicz, 2021). Occurrence of lymphadenopathia (Walsh-Messinger et al., 2021) and long-term persistence/repeated occurrence of elevated temperature and fever in post-acute period in COVID survivors (Goërtz et al., 2020), together with positive associations between severity of post-COVID neurological symptoms and elevated levels of pro-inflammatory markers (Lorkiewicz and Waszkiewicz, 2021; Sun et al., 2021) may speak in favor of the role of immune deregulation which may be coupled with immune-mediated adaptation of CNS to inflammatory processes linked with post-acute COVID-19.

However, future research investigating the relation between immune profiles, clinical representations and neuroimaging data is necessary to distinguish between the effects of immune-mediated adaptation to CNS and damage to neural tissue caused by excitotoxic effects of maladaptive immune responses to Sars-CoV-2. Furthermore, it is possible that pathogenesis of COVID-19-related neurological complications caused by immune-mediated processes can differ with regard to sex and age due to age and sex-dependent different patterns of immune responses to acute Sars-CoV-2 infection. In women, there was found a more robust response of T-cells to Sars-CoV-2 infection and worse disease outcome was associated with the higher levels of innate immune cytokines (Takahashi et al., 2020). On the other hand, men were found to display the greater activation of innate immunity and worse disease outcome was associated with poor response of T cells (Takahashi et al., 2020). Poor T-cells were also negatively correlated with the age of the patients (Takahashi et al., 2020). Furthermore, new Sars-CoV-2 variant (mutant S24L), which induces more active responses of immune system to acute Sars-CoV-2 infection in females than in males, has been discovered (Wang et al., 2021). Therefore, it is possible that COVID-19-related neurological complications caused by immune-mediated adaption of CNS and/or immune-mediated damage to neural tissues in acute and/or post-acute phase of COVID-19 may be qualitatively and quantitatively different in females than in males.

### COVID-19 Related Malfunctions of Extra-Neural Organs

Apart from COVID-19-related pathological processes taking place in CNS, COVID-19-related alteration of proper functioning of extra-neural tissues and organs might play an important role in exacerbation of neurological disturbances. For instance, endocrine glands producing hormones such as cortisol and sex hormones are heavily integrated in regulation of CNS functioning *via* hypothalamic-pituitary axis (HPA) (Pittman, 2011). Cortisol depletion caused by intense and/or long-term inflammatory processes might serve as an example for extra-neural etiologies of COVID-19-related neurological disturbances. Possibly due to the fear of death and/or neuro-endocrine-immune regulatory processes in acute COVID-19, high concentrations of cortisol levels were documented (Tan et al., 2020). Possibly because of cortisol depletion in acute COVID-19, in post-acute COVID-19 condition, hypocortisolism linked with neurological disturbances, such as inability to concentrate and fatigue, were already reported (Ayat et al., 2021). Possible extraneural etiology of long-term post-COVID neurological symptoms may be at least partially supported by documented long-lasting dysfunctions of one or more organs in COVID-19 survivors (Dennis et al., 2021; Selvaraj et al., 2021). Another example of hypothetical causal link between the injury to extra-neural tissues caused by COVID-19 and neurological problems, might be associated with the cases of COVID-19-related pathophysiological processes in neuromuscular junction. Damage to muscle, which seems to be vulnerable target to Sars-CoV-2 infection (Rudroff et al., 2021) due to sensitivity to some pro-inflammatory markers (Tay et al., 2020) and the presence of ACE2 receptors (Ferrandi et al., 2020), may lead to decreased motor performance resulting in peripheral fatigue (Hunter, 2018; Rudroff et al., 2020). Neural and muscular parts of neuromuscular junction have reciprocal trophic effects on each other (Guth, 1968; Funakoshi et al., 1995). Consequently, muscle dysfunction may cause secondary dysfunction of adjacent neural structures due to the trophic influences of muscle on motor unit (Funakoshi et al., 1995). The lack of muscular trophic factors for motor unit might trigger tertiary dysfunctions of higher-order adjacent brain systems leading to the exacerbation of central fatigue and/or other neurologic complications.

### Psychological Factors

Psychological factors may represent one of the important mechanisms responsible for the exacerbation and/or maintenance of a various neurological COVID-19-related symptoms in acute and post-acute period of COVID-19. In acute COVID-19, depression, fears and social isolation can worsen clinical condition and lead or contribute to exacerbation of a various neurologic and psychiatric conditions, such as anxiety and post-traumatic stress disorder (PTSD) (Fu et al., 2021; Samkaria et al., 2021) and fatigue (Morgul et al., 2021). Psychological stress related to acute COVID-19 is likely to be caused by the fear of death, fear of infecting family and other people and by social isolation due to quarantine.

In relation to post-acute COVID-19 period, psychological factors are also very likely to play an important role in exacerbation of new post-COVID-19 symptoms and/or maintenance or worsening of the actual ones. This notion can be at least partly supported by documented effects of psychological factors such as stress, fears and anxiety on exacerbation of fatigue in recovering COVID-19 survivors (Rudroff et al., 2020). Psychological stress related to post-acute COVID-19 is likely to be present due to multiple causes such as fear of the unknown health consequences of that disease, fear of re-infection and fear of decreased general functioning in daily life due to the reconvalescent period.

Contribution of psychological factors to neurological consequences of COVID-19 can be at least partly supported by the promising outcomes of psychotherapy (Hu et al., 2020; Yang et al., 2020) and positive thinking therapy (Alhempi et al., 2021) which were both demonstrated to be capable of reduction of some physical and psycho-social problems caused by COVID-19. Evidence speaking in favor of the existence of the link between psychological factors and COVID-19-related long-lasting disturbances comes from the study done by Thye et al. (2022). In that study, positive association between pre-existing worsened psychological status and increased risk of exacerbation of long-term post-COVID disturbances was documented (Thye et al., 2022).

### Mutual Co-occurrence and Interference Between Multiple Post-COVID-19 Neurological Symptoms

COVID-19-related neurological complications may occur as a single isolated symptom or the co-occurrence of multiple symptoms (Pilotto et al., 2021). Simultaneous co-occurrence of multiple neurological problems might possibly implicate the following two scenarios: (1) multiple neurological complications might have occurred approximately at the same time as a consequence of the COVID-19-related direct or indirect damage to the brain structure/s which is/are naturally responsible for multiple functions. For instance, amygdala, which is responsible for the functions such as emotional regulation (Kim et al., 2011), autonomic regulation (Reisert et al., 2021) and is also involved in olfactory functions (Kevetter and Winans, 1981; Zald and Pardo, 1997; Buchanan et al., 2003), was repeatedly found to exhibit abnormalities in post-COVID-19 condition (Douaud et al., 2021; Guedj et al., 2021a; Morand et al., 2021; Sollini et al., 2021). (2) Secondly, some complications may develop earlier and they may secondarily trigger other ones. That may be one of the possible explanations of why some disturbances linked with COVID-19 occur at the early phases of this infection whereas other problems develop after a longer elapsed time (Delorme et al., 2021; Fernández-De-las-Peñas et al., 2021a). Therefore, co-occurrence and co-interaction of multiple co-morbidities may lead to exacerbation of new co-morbidities and/or long-lasting maintenance of them. For instance, dizziness, migraines and anxiety found in post- COVID patients were found to precede the later development of seizures (Park et al., 2021). In another study, the severity of headaches in acute COVID-19 infection was positively associated with the severity level of post-COVID-19 fatigue and headaches (Fernández-De-las-Peñas et al., 2021b). There are some studies finding a positive correlation between some post-COVID-19 neurological problems, such as between fatigue and anxiety (Townsend et al., 2021), fatigue and anhedonia (El Sayed et al., 2021). These findings may implicate some kind of interdependency between these post-COVID-19 complications. This hypothesis may be at least partially supported by the fact that some neurological complications are likely to co-occur, for instance, depression, fatigue and pain are frequently present in people suffering from chronic fatigue syndrome (Komaroff and Lipkin, 2021) and from multiple sclerosis (Workman et al., 2020). The severity of these symptoms may be cross-correlated with each other (Bould et al., 2013). Such co-occurrence of some particular neurological complications in a various pathological conditions may speak in favor of their casual interrelatedness irrespective of their etiology. For instance, the positive correlation between the level of fatigue and the level of anxiety found in post-COVID-19 condition (Townsend et al., 2021) may indicate that the level of fatigue is casually dependent on the level of anxiety and vice versa. In other words, anxiety may generate fatigue due to permanent anxiety-related hyperarousal and conversely, a permanent fatigue may represent a source of anxiety as fatigued individuals may feel anxious of finding the way of overcoming their burden of fatigue.

Based on these findings, it can be postulated that mutual co-occurrence and interference between multiple COVID-19 related symptoms can be responsible for the exacerbation and/or maintenance of another disturbances during both, acute and post-acute period of COVID-19.

It can be seen that our proposed factors possibly responsible for exacerbation and long-term persistence of COVID-19-related neurological conditions are likely to overlap with each other and they can probably co-exist together. Also, it is necessary to bear in mind that there are probably no strict boundaries between damages to CNS caused by Sars-CoV-2, long-term recovery of damaged neural tissues, immune-mediated adaption of CNS to inflammatory state, COVID-19-related damage to extraneural tissues, psychological factors and co-interaction and mutual interference between multiple post-COVID symptoms. For that reason, our proposed classification of these factors is likely to be a simplification and approximation of the reality.

To summarize this chapter, the following Table 1 is supposed to summarize all 6 aforementioned proposed pathophysiological mechanisms responsible for COVID-19-related neurological problems in relation to acute and post-acute phase of Sars-CoV-2 infection.

**TABLE 1:** Summarization of possible manifestations of pathophysiological mechanisms responsible for occurrence of COVID-19-related neurological problems in relation to acute and post-acute phase of Sars-CoV-2 infection.

Possible pathophysiological mechanisms responsible for occurrence of COVID-19-related neurological problems	Possible manifestation of the particular pathophysiological mechanism in ACUTE Sars-CoV-2 infection	Possible manifestation of the particular pathophysiological mechanism in POST- ACUTE Sars-CoV-2 infection
1. Direct damage of Sars-CoV-2 to neural tissue	Yes	Yes
2. Indirect damage of Sars-CoV-2 to neural tissue	Yes	Yes
3. Long-term recovery of damaged neural tissues	It probably does not manifest in the early phases of the acute infection. Rather, it is likely to start to manifest in its later phases (sub-acute phase)	Yes
4. COVID-19-related dysfunction of extraneural tissue	Yes	Yes
5. Psychological factors	Yes	Yes
6. Mutual co-occurrence and interference between multiple post-COVID-19 neurological symptoms	Yes	Yes

## Post-COVID-19 Neurological Complications From the Perspective of Dynamic System Theory

Investigation of the link between behavior of biological organisms and dynamic system theory has recently become a widely investigated topic of interest (Ros et al., 2014; Zimmern, 2020; Burrows et al., 2021; Nemzer et al., 2021). Its outcomes bring many valuable papers integrating concepts from neuroscience, physics and information theory (Stam, 2005). Based on the current knowledge related to post-COVID-19 neurological complications and system’s behavior, we speculate that post-COVID-19 complications may be understood and viewed as a consequence of deregulation and/or disruption of system parameters and emergence of maladaptive attractors turning brain into operating out of its critical regime.

First of all, it is necessary to define the basic terms from dynamic system theory such as system parameters, attractors and criticality.

### System Parameters

System parameters refer to the term of system attributes whose values determine system’s behavior (Stam, 2005). For instance, physical quantities such as temperature, volume and pressure are examples of system parameter. The modulation of the system parameters values cause changes in system’s behavior (Hesse and Gross, 2014). Analogically, biological systems possess an enormous number of hormones, enzymes, neurotransmitters and cytokines which all can be viewed as system parameters as alterations in their concentrations and/or activity will cause significant changes in the behavior of the organism. For example, gamma-aminobutyric acid (GABA) represents the major inhibitory neurotransmitter in CNS. It participates in a variety of functions such as regulation of sleep (Watanabe et al., 2002), seizure prevention (Möhler, 2006) and others (Möhler, 2006; Hepsomali et al., 2020). Pathological enhancement of GABAergic activity can cause abnormal somnolence (Trotti et al., 2015) whereas over-reduction of GABA levels and/or its receptors can cause exacerbation of epileptic seizures (Schiene et al., 1996). In relation to post-COVID-19 problems, post-COVID-19 central fatigue was proposed to stem from COVID-19-related alterations of the levels of neurotransmitters such as serotonine and dopamine (Rudroff et al., 2020). Although, to the best of our knowledge, the relationship between the levels of neurotransmitters and post-COVID-19 disturbances has not been investigated yet, it is possible that neurotransmitters and their levels might represent important system parameters involved in determination of various post-COVID-19 symptoms and their dynamics. Based on the findings from the studies dedicated to the investigation of post-COVID-19 complications, we postulate that post-COVID-19 complications may portray manifestations of pathological alterations of system parameters, emergence of new pathological attractors which are responsible for disruption of homeostasis and consequent disability to operate in/near the critical point of the system. In relation to acute COVID-19 and post-COVID-19 condition, levels of inflammatory cytokines and leukocytes seem to represent important system parameters influencing the system’s behavior. Increased pro-inflammatory cytokines and altered levels of leukocytes plus levels of lymphopenia were associated with increased risk of exacerbation of COVID-related neurological complications (Sun et al., 2021) and with higher severity of them (Sun et al., 2021; Visvabharathy et al., 2021). Immunotherapy has been found to be effective in normalizing immune profile and improvement of post-COVID neurological pathological conditions (Carroll et al., 2020; Sangare et al., 2020). It is currently unknown how many cytokines, enzymes, neurotransmitters and hormones are directly or indirectly affected by COVID-19 and how such alterations in the levels of these system parameters and their activity would manifest in acute and post-acute phase of COVID-19.

### Attractors

When the change of system parameters is very rapid and/or intense, new attractors may emerge (Stam, 2005). Attractor of the system represents the most probable state which the system has the greatest tendency to evolve to Stam (2005). According to thermodynamics laws, each system has a tendency to evolve into the state requiring the minimal energy cost (Haddad, 2013). Within the reign of living organisms, the ideal state, which biosystems try to evolve to, should meet these two following conditions: (1) there should be the minimal energy cost and (2) it should be the most efficient for a proper functioning of the whole system (Johnson, 1992).

New pathological attractors, emerging from COVID-19-related structural and functional abnormalities documented in a various brain regions, might be responsible for making brain to turn into conditions of post-COVID-19 complications such as seizures, dizziness, headaches etc.

From neurological point of view, in relation to excitatory or inhibitory manifestation of the symptoms, there are two types of neurological symptoms- so-called positive and negative symptoms (Berman et al., 1997; Brown and Pluck, 2000; Strauss and Cohen, 2017). Positive symptoms are manifested as the states of over-increased and/or disorganized brain functions, for example, pain, anxiety and delirium may belong to this category (Berman et al., 1997). On the other hand, negative symptoms manifest as the occurrence of conditions characterized by decreased level/loss of the brain functions, such as loss of memory (Strauss and Cohen, 2017). In the connection to post-COVID-19 neurological complications, both types of symptoms can be found within the realm of post-COVID-19 symptoms. Positive symptoms might include conditions such as headaches, seizures, pain, anxiety and delirium. Negative symptoms might involve conditions such as memory loss, hypo/anosmia and hypo/ageusia. Occurrence of positive and negative symptoms or the co-occurrence of both might theoretically correspond to neurological profiles of misbalance between activation levels within the particular brain areas affected by COVID-19. In other words, some areas may get hyperactivated whereas other ones hypoactivated and the mutual misbalance of their activity levels may generate various pathological conditions.

### Criticality

Critical behavior of the system refers to the state of the system in which there is a balance between order and entropy (Hellyer et al., 2014). In this state, the system is the most capable of switching between a multiple different states managing to benefit from each of these states but at the same time the stability of the system is maintained (Stam, 2005; Pastukhov et al., 2013). In relation to critical behavior of CNS, homeostatic plasticity has been proven to play a very important role in critical behavior (Ma et al., 2019) as it tunes CNS functioning to the state of balance between excitatory and inhibitory influences (Turrigiano and Nelson, 2000). When there comes to disruption of homeostatic regulation of biosystem, the system is likely to turn into either supercritical or subcritical regimes (Stam, 2005; Ma et al., 2019). Supercritical regime refers to the condition in which there is almost no order and entropy becomes dominant (Stam, 2005; Freyer et al., 2011). The system loses its stability, its behavior becomes chaotic and disorganized (Stam, 2005; Freyer et al., 2011) and there comes to hyper-responsiveness due to increased but chaotic information transfer (Poel et al., 2021). On the other hand, subcritical regime refers to the state in which there comes to maximal level of order and minimal level of entropy. In this regime, the system becomes rigid and unresponsive to stimuli due to hyper-organization which prevents from flexibility (Stam, 2005; Freyer et al., 2011). Post-COVID-19 coma was found to be characterized by dominant large-scale prevalence of alpha activity in EEG with minimal or no-reactivity to amplitude modulation (Koutroumanidis et al., 2021) which might be hallmarks of the manifestation of subcritical regime. On the other hand, possible occurrence of supercritical regime or the possible risk factor for being likely to turn into operating in supercritical regime, might be hypothesized to occur in COVID-19 survivors with PTSD in which altered organization of dynamic functional connectivity has been found (Fu et al., 2021). Reduction of node strengths and their degrees and efficacy, which was found in neural networks of these COVID survivors (Fu et al., 2021), may be linked with increased level of entropy or with higher risk of increased entropy levels causing consequent increase of information noise in neuronal communication.

However, so far, our proposed perspective of viewing post-COVID-19 complications from the perspective of dynamical system theory suffers from the lack of studies investigating the link between post-COVID-19 complications and critical behavior of the systems. We believe that future studies, using mathematical analytical approaches for studying links between neuroimaging data and clinical manifestations of post-COVID complications, may shed more light on this issue. Nevertheless, based on the current clinical and neuroimaging studies, we propose that post-COVID-19 complications are linked with maladaptive alterations of system parameters and emergence of new pathological attractors responsible for turning the brain to operate far from its critical (optimal) state.

For that reason, we propose that therapy targeting post-COVID-19 complication should be capable of elimination of pathological attractor, renormalization of system parameters and tuning of system to operate in its critical regime.

## Biofeedback Therapy as a Potential Treatment for Post-COVID Condition

So far, post-COVID-19 complications have been treated pharmacologically (Blitshteyn and Whitelaw, 2021; Pattnaik et al., 2021), immunologically (Carroll et al., 2020; Sangare et al., 2020), by oxygen therapy (Kamal et al., 2021) and by rehabilitation exercises (Spielmanns et al., 2021). In relation to non-invasive brain modulation techniques, potential benefits of transcranial magnetic and transcranial direct current stimulation have been proposed for the treatment of COVID-19-related complications (Baptista et al., 2020). Transcranial direct current stimulation has been already documented to be effective for reduction of subjective fatigue in COVID-19 survivors (Workman et al., 2021).

Biofeedback (BFB) represents non-invasive therapy based on self-regulation of one’s internal state based on delivering information of biosignal changes to the participant (Gruzelier, 2014; Enriquez-Geppert et al., 2017). Once the amplitude of biosignal (e.g., EEG activity) reaches the level, which is at least as high as the level set by BFB therapist; the participant receives auditory and/or visual feedback. Due to repeated receiving of these feedbacks, the brain is able to associate these feedbacks with the underlying mental and psycho-physiological states linked with rewarded activity. Consequently, due to associative learning, it is becoming easier and easier for the participant to reach the states associated with increased levels of rewarded targeted activity (Gruzelier, 2014; Enriquez-Geppert et al., 2017). Biosignal modalities may include heartbeats patterns, electroencephalogram (EEG) changes, blood oxygen-dependent changes and others (Gruzelier, 2014; Collura, 2017; Watanabe et al., 2017; Lehrer et al., 2020). Biofeedback modalities, which are targeted to modify brain biosignals, such as EEG or blood oxygen-dependent changes in brain, are termed neurofeedback (NFB). Rewarded levels of targeted biosignals are set in accordance with the scientific and clinical knowledge related to the particular functions of the biosignals and the particular values of these biosignals documented in healthy and pathological conditions (Gruzelier, 2014; Collura, 2017; Enriquez-Geppert et al., 2017). BFB has been found to be capable of improving various neurological problems such as headaches (Walker, 2011), insomnia (Hammer et al., 2011), depression (Paquette et al., 2009; Tsuchiyagaito et al., 2021), fatigue (Hammond, 2001), epileptic seizures (Walker and Kozlowski, 2005) which overlap with the symptoms frequently found in post-COVID-19 conditions as well (Carroll et al., 2020; Dono et al., 2021; Guedj et al., 2021b; Kincaid et al., 2021; Lorkiewicz and Waszkiewicz, 2021; Park et al., 2021). Furthermore, NFB was found to be effective for treatment of chronic fatigue syndrome (CFS) (Hammond, 2001) and multiple sclerosis (MS) (Ayache et al., 2021) which both share similar symptoms to post-COVID-19 complications, such as pain, fatigue and depression (Workman et al., 2020; Komaroff and Lipkin, 2021; Wostyn, 2021). In addition, as well as in case of post-COVID-19 syndrome, both CFS and MS may stem from infectious agents (Steiner et al., 2001; Ghasemi et al., 2017; Komaroff and Lipkin, 2021). For that reason, we believe that BFB may represent a promising therapy for post-COVID-19 complications. Our proposal might be supported by the fact that NFB has been found to initiate micro-structural changes of white and gray matter associated with the improvement of functions of the trained brain areas (Ghaziri et al., 2013). In addition, NFB-induced behavioral improvements of brain functions were repeatedly documented to have a long-term persistence (Kerson et al., 2009; Alexeeva et al., 2012; Van Boxtel et al., 2012; Mottaz et al., 2015). Neurofeedback (NFB) was also shown to be capable of tuning EEG oscillations to operate near the state of criticality (Zhigalov et al., 2016; Ros et al., 2017) which may be possibly the hallmark of NFB capability to help to restore system behavior to operate in its optimal (critical) state which may be possibly of particular significance for post-COVID-19 complications. Furthermore, NFB therapy was also associated with increased levels of brain derived neurotrophic factor (Markiewicz and Dobrowolska, 2020), which is involved in regulating homeostatic plasticity (Turrigiano and Nelson, 2000), and which plays an essential role for successful maintenance of critical regime of brain dynamics (Ma et al., 2019).

In the following sections, we will discuss a potential role of biofeedback as a possible therapy for the treatment of post-COVID-19 complications. The proposed biofeedback modalities involve: (1) fMRI-based neurofeedback, (2) QEEG-based neurofeedback, (3) Othmer’s method of rewarding individual optimal EEG frequency and (4) HRV biofeedback.

### fMRI-Based Neurofeedback

**FIGURE 1:**
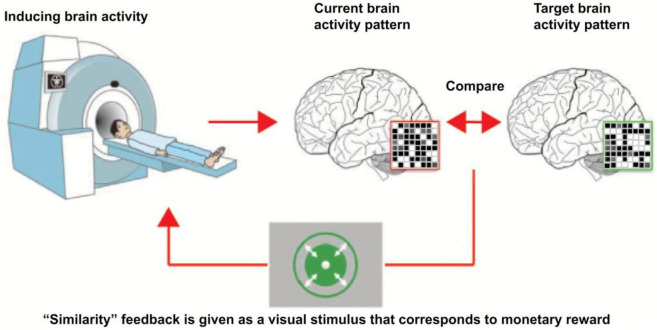
Underlying principles of fMRI-based neurofeedback. This figure describes the underlying principles of fMRI-based neurofeedback. Magnetic activity of the participant head is monitored. Neurofeedback system constantly compares the current brain activity pattern with target brain activity pattern. As soon as the current brain activity pattern is sufficiently close to the target brain activity pattern, neurofeedback system generates rewarding feedback for the participant [taken from Yamada et al. (2017), taken with the permission of the author].

fMRI neurofeedback (fMRI-NFB) is based on increasing or decreasing amplitude of fMRI signal which is averaged across the regions of interest (ROIs) in a brain (Watanabe et al., 2017). fMRI-NFB signal consists in the extraction of online blood level oxygen dependent (BOLD) signal (Haller et al., 2013; Watanabe et al., 2017). There are two major approaches of fMRI-NFB: The training of a particular targeted area, and the training of a neural network which may consist of two or more brain regions (Haller et al., 2013; Watanabe et al., 2017). The former type of fMRI-based NFB consists in increasing or decreasing the activity in the particular ROI, whereas the latter one relates to the modulation of activity within 2 or more brain areas (Watanabe et al., 2017). FMRI-based NFB was found to be capable of improving various neuro-pathological conditions (Bauer et al., 2020; Tsuchiyagaito et al., 2021). FMRI-based NFB has been found to be capable of altering connectivity strength in a targeted neural network (Haller et al., 2013; Koush et al., 2013). This kind of NFB modality can promote recovery from a neurological disorders accompanied with abnormalities in brain connectivity (Haller et al., 2013). The following Figure 1 illustrates the underlying principles of fMRI-based NFB.

In post-COVID-19 condition, various abnormalities have been found in a various brain regions including damage to gray and white matter, abnormal hypometabolism and hypermetabolism of a various brain areas and impairment in brain connectivity (Carroll et al., 2020; Sangare et al., 2020; Dono et al., 2021; Douaud et al., 2021; Fu et al., 2021; Guedj et al., 2021a; Morand et al., 2021; Younger, 2021).

Since fMRI-based NFB can modulate brain network abnormalities and down-regulate abnormalities even in subcortical areas (Haller et al., 2013; Tsuchiyagaito et al., 2021) which are frequently found to exhibit functional and structural abnormalities in post-COVID condition (Guedj et al., 2021a; Sollini et al., 2021; Younger, 2021), we propose that fMRI-NFB may be beneficial for the treatment of post-COVID-19 conditions accompanied by aberrant patterns of brain activity. FMRI-based NFB for up-regulation of activity of target brain area/areas might be beneficial for hypometabolic condition in which activity in affected areas is reduced. On the other hand, down-regulation of brain activity might be beneficial for conditions of hypermetabolism and hyperconnectivity which can also occur in post-acute COVID period (Fu et al., 2021; Kincaid et al., 2021). In case of focal brain damage, patients might be likely to benefit from fMRI-NFB targeted to train activity in single ROI. On the contrary, connectivity-based NFB-fMRI might be useful for abnormal hypo/hyperconnectivity within multiple brain regions.

The following mentioned hypometabolic and hypermetabolic brain areas correlated with a various post-COVID-19 disturbances may serve as potential therapeutic target areas to fMRI-based NFB. Higher severity of hyposmia was positively associated with hypometabolism in bilateral gyrus rectus, medial frontal gyri and right middle temporal cortex (Morbelli et al., 2022). Positive correlation was documented between fronto-parietal hypometabolism and impairment of memory and executive functions measured by Montreal Cognitive Assessment performance (Hosp et al., 2021) which is consistent with the functional involvement of fronto-parietal network in executive functions (Sauseng et al., 2005). The findings documented in Hosp et al. (2021) study are at least partly consistent with another study which documented positive link between impairment in cognitive performance and reduction of metabolic activity in fronto-parietal network and temporal areas in COVID-19 survivors (Blazhenets et al., 2021). Hypometabolism in the right temporal lobe was also found to be positively correlated with pain, insomnia and longer duration of initial infectious symptoms (Guedj et al., 2021b). Positive linkage was also observed between hypometabolism of frontal areas and the presence of pain and high blood pressure (Guedj et al., 2021b), possibly indicating disturbances of central autonomous nervous system. In addition, cerebellar hypometabolism was positively correlated with increased number of various post-COVID-19 complaints (Guedj et al., 2021b). Apart from documented positive correlations between increased number and severity of post-COVID-19 disturbances, there are also existing reports of existing link between brain hypermetabolism and severity of post-COVID-19 problems. Namely, positive link between the levels of metabolism in orbitofrontal and parietal cortices and greater severity and longer duration of COVID-19-related dysosmia was observed (Niesen et al., 2021). However, apart from documented positive correlations between severity of post-COVID-19 symptoms and the level of decreased or increased metabolism in a various brain regions, there are also existing reports of more complex relationships between brain metabolism and severity of post-COVID-19 disturbances. The study done by Du et al. (2022) discovered positive correlation between amplitude of low frequency fluctuations in the left caudate nucleus and severity of sleep problems measured by Athens Insomnia Scale (Du et al., 2022). For that reason, advanced mathematical analysis of biosignals may be helpful to come up with successful NFB therapy for COVID-19 survivors in which positive association between neurological problems and complex brain metabolic patterns is present.

Nevertheless, fMRI-NFB was found to be capable of alteration of activity in other non-trained regions (Shibata et al., 2019) and therefore complex NFB outcomes might be expected. Our proposal of possible benefits from fMRI-based NFB on post-COVID neurological symptoms may be at least partly supported by the findings from other studies discovering that fMRI-based NFB is capable of improving various neurological disturbances that overlap with post-COVID-19 symptoms (Patel et al., 2020; Barizien et al., 2021; El Sayed et al., 2021; Fernández-De-las-Peñas et al., 2021c; Lorkiewicz and Waszkiewicz, 2021; Townsend et al., 2021; Tsuchiyagaito et al., 2021). Furthermore, fMRI-based NFB was demonstrated to improve neuro-immune regulatory pathway by up-regulation of activity in left amygdala (Tsuchiyagaito et al., 2021). Such fMRI-NFB-based improvement in neuro-immune regulation might be beneficial for post-COVID-19 patients suffering from long-term inflammation and deregulation of immune system.

Limitation of our proposal of suitability of fMRI-based NFB for treatment of a post-COVID-19 neurological complication may consist in several aspects. First, we do not know whether post-COVID-19 neurological problems would successfully respond to that kind of treatment. Second, it is not known yet whether and which neurological post-COVID-19 symptoms are causally associated to the brain abnormalities found in neuroimaging studies done on COVID survivors. The last but not least, occurrence of post-COVID-19 neurological complications may not accompanied by abnormalities in brain structures (Fischer et al., 2020; Lim et al., 2020; Paterson et al., 2020). For that reason, COVID-19 survivors suffering from neurological complications having no structural brain abnormalities might be more likely to benefit from other types of NFB modalities.

### Quantitative Electroencephalogram-Based Neurofeedback as a Potential Training for Electroencephalogram Abnormalities in Post-COVID-19 Condition

Quantitative EEG (QEEG) is based on enhancing or inhibiting certain EEG activity which is selected as a target NFB reward activity in accordance with pre-NFB EEG assessment (Collura, 2017). QEEG-based NFB protocols share a special narrative EEG databases containing statistically huge collection of EEG values of healthy people and EEG values of neuro-pathological condition as well as links between EEG patterns and various clinical conditions (Collura, 2017). Abnormal EEG activity may therefore represent a potential pathological attractor responsible for turning brain behavior into maladaptive regimes of operating far from optimal set-point. QEEG-based NFB is used for normalizing the pathological EEG activity by NFB-induced reduction of abnormally high coherence/amplitude and for up-regulation of abnormally low EEG amplitude/coherence values (Walker, 2010; Collura, 2017). Numerous QEEG NFB studies showed that QEEG-based NFB down-regulation of abnormal EEG patterns is connected with various behavioral improvements such as cessation or reduction of epileptic seizures (Walker and Kozlowski, 2005), improvement in cognitive abilities (Kerson et al., 2009) and others (Hammond, 2001; Paquette et al., 2009). NFB-induced reduction of aberrant EEG activity can be associated with the level of behavioral improvement (Hammer et al., 2011). These findings may speak in favor of ability of QEEG-based NFB to restore homeostatic set-point by teaching brain to operate in regimes which are far away from pathological regimes generated from pathological attractors linked with aberrant EEG activity. There have been documented a numerous aberrant pathological patterns found in a various neuropathological conditions (Díaz et al., 1998; Hammer et al., 2011; Walker, 2011) including post-COVID-19 condition as well (Kincaid et al., 2021). To the best of our knowledge, no study has been done to investigate the potential link between the severity of post-COVID-19 neurological complications and EEG profile yet. However, in acute COVID-19 condition, in which pathological EEG activity can occur (Flamand et al., 2020; Pellinen et al., 2020; Kubota et al., 2021), positive correlation between the level of EEG slowing and severity of clinical symptoms of the patients has been already documented (Pasini et al., 2020). These findings indicate that the investigation of EEG profiles and their links with clinical symptoms in post-COVID conditions may bring fruitful and valuable findings which may serve as a therapeutic target. We propose that QEEG-based NFB may serve as a beneficial non-invasive therapy for post-COVID neurological complications which are accompanied with abnormal EEG profiles. This proposal is based on the findings of multiple QEEG-NFB studies which managed to improve similar pathological conditions as those that frequently occurs in post-COVID period such as epileptic seizures (Wyler et al., 1976; Walker and Kozlowski, 2005; Walker, 2011) depressions (Paquette et al., 2009), fatigue (Hammond, 2001) and insomnia (Hammer et al., 2011) by NFB-induced reduction of pathological EEG associated with these conditions. Furthermore, level of behavioral improvement was repeatedly found to correlate with the level of NFB-induced reduction of target aberrant EEG activity (Paquette et al., 2009; Hammer et al., 2011). These positive associations might be in line with positive associations found between the severity of some particular neurological symptoms and the level of aberrant EEG activity (Díaz et al., 1998; Askew, 2001). So far, it is unknown whether post-COVID-19 neurological complications are associated with some specific EEG patterns. So far, various abnormal EEG patterns were repeatedly found in COVID-19 survivors suffering from *de novo* seizure conditions (Carroll et al., 2020; Dono et al., 2021; Kincaid et al., 2021). However, to the best our knowledge, no study has investigated the link between the severity of pathological EEG and severity of seizures yet. Furthermore, to the best of our knowledge, it is unknown by now if there is a specific EEG pattern found in other post-COVID-19 neurological complications, such as migraines, dizziness, anxiety, depression etc. and whether there exists a causal link between these post-COVID-19 problems and particular EEG profile. Apart from the lack of this knowledge, post-COVID-19 neurological complications do not have to be always accompanied with abnormalities in EEG profile (Lim et al., 2020). For that reason, other NFB modalities might be more suitable for such cases.

### Othmer’s Method of Training of Individual Optimal Electroencephalogram Frequency

Othmer’s method of rewarding optimal training EEG frequency is based on individual adjustment of NFB-rewarded EEG frequency band width (Othmer and Othmer, 2017; Othmer, 2020). Optimal rewarded EEG frequency (ORF) is associated with mental state during which one feels calm, alert and relaxed and it is connected with the state of so-called optimal arousal (Othmer and Othmer, 2017; Othmer, 2020). On the other hand, too high NFB-rewarded frequency is linked with irritating symptoms such as headaches, anxiety, onset insomnia, and nightmares which are the signs of high arousal (Othmer and Othmer, 2017; Othmer, 2020). This may be in accordance with positive linkages between excessive high beta activity and neurological disturbances such as anxiety (Walker, 2010; Díaz et al., 2019), headaches (Walker, 2011) and insomnia (Perlis et al., 2001; Hammer et al., 2011). On the contrary, hypoarousal (low arousal) occurs when the training NFB frequency is too low and it results in symptoms such as somnolence, fatigue, apathy and sadness (Othmer and Othmer, 2017; Othmer, 2020). This notion might be in line with the prevalence of low frequency activity in conditions such as fatigue (Klimesch, 1999; Lafrance and Dumont, 2000) and somnolence (Hammer et al., 2011). ORF and the corresponding optimal arousal can be analogous to Yerkes-Dodson model of inverted U-like relationship between the arousal level and level of learning and Hebb’s model of U-like relation between level of arousal and level of cognitive performance (Teigen, 1994). In both of these models, low arousal is linked with the states of coma or sleep coupled with low level of behavioral performance whereas high arousal levels are associated with the states of anxiety and panic and are also linked with lower levels of behavioral performance. On the other hand, moderate levels of arousal are associated with the highest levels of behavioral cognitive performance (Teigen, 1994). Hebb’s and Yerkes-Dodson’s models of relationship between behavioral performance and levels of arousal may be also reminiscent of system’s behavior according to dynamical system theory. In this analogy, critical system’s behavior would be linked with rewarding of individual ORF, whereas subcritical regime might be the result of rewarding too low EEG frequency and supercritical regime might occur as a result of NFB training of too high EEG frequency.

Post-COVID-19 complications involve symptoms such as headaches, anxiety and insomnia (Fernández-De-las-Peñas et al., 2021c,d; Townsend et al., 2021) which are typical for presence of state of high arousal (Hammer et al., 2011; Walker, 2011; Othmer and Othmer, 2017; Díaz et al., 2019; Othmer, 2020) as well as symptoms reminiscent of low arousal such as somnolence and fatigue (Othmer and Othmer, 2017; Othmer, 2020). Therefore, we propose that NFB training of ORF may lead to normalization of arousal level leading to improvement of the aforementioned post-COVID-19 symptoms. As post-COVID-19 complications may not be necessarily accompanied with any structural and/or electrophysiological brain abnormalities (Fischer et al., 2020; Lim et al., 2020; Park et al., 2021), the advantage of this Othmer’s method of ORF would consist in its methodology that does not rely on neuroimaging data of the patient. Instead, individualized NFB protocol is entirely based on the symptomatology of the patient (Othmer and Othmer, 2017; Othmer, 2020). Othmer’s method of ORF has been successfully applied on a variety of neurophysiological problems (Putman, 2004; Sime, 2004; Legarda et al., 2011; Carlson and Ross, 2021) which greatly overlap with post-COVID-19 neurological complications (Caronna et al., 2021; Fernández-De-las-Peñas et al., 2021b; Pilotto et al., 2021).

In relation to possible limitations, we do not know whether post-COVID-19 symptoms would successfully respond to this kind of treatment compared to analogical symptoms of different etiologies which have been already found to be successfully treated by Othmer’s method of ORF. Another important notion that should be taken into consideration is the possibility that some symptoms portraying the characteristics of low arousal might be in fact caused by high arousal and vice versa, symptoms reminiscent of high arousal may be in fact the consequence of low arousal. For instance, fatigue can be the result of high arousal-like states such as insomnia (Hammer et al., 2011) and/or anxiety (Díaz et al., 2019) and in this case lowering training EEG frequency instead of its increase can be the successful strategy. In post-COVID-19 condition, significant positive correlation has been found between the level of fatigue and anxiety (Townsend et al., 2021) which may indicate mutual interrelatedness of these symptoms and their potential causal inter-relation and this kind of inter-relation may be that case in one needs to be very careful in proper classification of symptoms resulting from high or low arousal condition. All in all, we believe that Othmer’s method may represent a promising therapy for post-COVID-19 neurological complications.

### Heart Rate Variability-Based Biofeedback

Heart rate variability-based biofeedback (HRV-based BFB) is based on feeding back heart rate data to the participant during breathing, such that the participant tries to maximize his/her respiratory sinus arrhythmia (RSA). When breathing, RSA is the heart activity corresponding to the changes of heart rate which is influenced by breathing such that heart rate increases during inspiration and decreases during expiration and gas-exchange efficacy in lungs becomes maximized when breathing and heart oscillations become coherent (Gevirtz, 2013; Lehrer et al., 2020). During HRV-based BFB training, RSA oscillations become more simple and sinusoidal which is often achieved within a several of minutes, even though the participant is completely naive to HRV-based BFB treatment (Gevirtz, 2013; Lehrer and Gevirtz, 2014). RSA is controlled by parasymphatetic autonomous nervous system (PANS) (Lehrer and Gevirtz, 2014; Lehrer et al., 2020). These postulates are also supported by studies which managed to find greater baroreflex after (Lehrer et al., 2003; Hassett et al., 2007) and/or during HRV-based BFB (Lehrer et al., 2003) and higher HRV after BFB-HRV (Hassett et al., 2007; Lin et al., 2019) and during BFB-based HRV (Caldwell and Steffen, 2018) since both high HRV and baroreflex are markers of PANS (Cowley and Guyton, 1975; Swenne, 2013; Tracy et al., 2016; Beltrán et al., 2020). Additionally, HRV-based BFB was reported to decrease blood pressure and heart rate (May et al., 2019) which may also speak in favor of its capability to modulate ANS toward the greater influence of PANS. Activation of baroreflex and activation of vagal afferents *via* subdiaphragmatic breathing accompanying HRV-based BFB are proposed mechanisms of HRV-based BFB-mediated effects on central autonomous nervous system (CANS) (Lehrer and Gevirtz, 2014; Lehrer et al., 2020) *via* activation of nucleus tractus solitarii which regulates baroreflex (Takagishi et al., 2010; Lin et al., 2013) and is also responsible for transferring signals to higher-ordered CANS (Reisert et al., 2021). Based on these notions, we hypothesize that post-COVID-19 haulers, especially those suffering from dysautonomia, might benefit from HRV-based BFB treatment. First of all, this proposal is based on the significant overlap between post-COVID-19 symptoms and symptoms of other etiologies that were successfully treated by HRV-BFB (Karavidas et al., 2007; Reiner, 2008; van der Zwan et al., 2015; Windthorst et al., 2017; Lin et al., 2019). Secondly, since HRV-BFB was found to be capable of enhancing markers of PANS, such as increased HRV (Hassett et al., 2007; Caldwell and Steffen, 2018) and increased baroreflex (Lehrer et al., 2003; Hassett et al., 2007), post-COVID-19 patients with under-activated/inhibited PANS and/or over-activated sympathetic autonomous nervous system (SANS) might represent a suitable candidates for HRV-BFB treatment. This proposal might be at least partially supported by reported alterations in HRV pattern found between fatigued post-COVID-19 and non-fatigued post-COVID-19 participants (Barizien et al., 2021). Thirdly, successful acquisition of the proper breathing pattern leading to success in HRV-BFB training can help to restore better breathing habits leading to more effective gas exchange in lungs resulting in greater oxygenation facilitating regeneration and/or reparation of damaged tissue in post-COVID-19 condition. Therapy involving HRV-BFB training might be especially of a great significance to those people who display maladaptive breathing patterns and/or other breathing complications that often occur in post-COVID-19 condition (Becker, 2021). Last but not least, ANS is strongly connected with the proper functioning of immune system (Andersson, 2005; Hilderman et al., 2019). SANS is involved in driving pro-inflammatory changes whereas PANS mediates suppression of pro-inflammatory markers (Andersson, 2005; Hilderman et al., 2019). HRV-BFB was found to enhance resilience to inflammatory marker, particularly, to lipopolysaccharide (Lehrer et al., 2010). HRV-based BFB was documented to decline lipopolysaccharide-related attenuation effects on HRV showing neuro-immune-regulatory potential of HRV-based BFB which may be indicative of its potential therapeutic benefits for post-COVID-19 participants suffering from long-term deregulation of immune system (Lehrer et al., 2010).

Another supporting evidence of potential therapeutic effect of HRV-based BFB for post-COVID-19 haulers comes from neuroimaging studies demonstrating increases in blood flow in amygdala, hippocampus, gyrus cingulate plus greater connectivity between sub-cortical limbic areas and prefrontal cortex during HRV-based BFB (Mather and Thayer, 2018; Vaschillo et al., 2019). The aforementioned limbic areas hugely overlap with the areas which were repeatedly found hypo-metabolic in post-COVID-19 period (Guedj et al., 2021a; Morand et al., 2021; Sollini et al., 2021) and were associated with some post-COVID-19 neurological complications (Douaud et al., 2021; Sollini et al., 2021) and at the same time they belong to CANS (Reisert et al., 2021). For that reason, we suggest that HRV-based BFB might help to normalize activity in CANS and restore its underlying functions in post-COVID-19 condition.

However, it is necessary to mention that no link has been found/investigated between the levels of alteration in ANS markers and severity of post-COVID-19 complications yet. Also, the particular type of dysautonomia is likely to play a crucial role. For instance, dysautonomia exhibiting reduced SANS and/or over/activated PANS might not be as suitable candidate as dysautonomia with predominant SANS for HRV-BFB therapy in which PANS is stimulated. Despite these limitations, we believe that HRV-BFB is worth-investigating as a potential and promising therapy for post-COVID-19 complications.

To summarize our considerations of suitability of aforementioned biofeedback modalities for post-COVID-19 neurological symptoms with regard to the presence of electrophysiological and/or functional brain abnormalities, Table 2 is proposed.

**TABLE 2:** Suitability of biofeedback modalities for post-COVID-19 neurological symptoms with regard to the presence of electrophysiological and functional brain abnormalities.

Biofeedback modality	Necessity of the presence of structural or functional brain abnormalities documented by neuro-imaging methods
1. fMRI-based neurofeedback	Yes
2. QEEG-based neurofeedback	Yes
3. Othmer’s neurofeedback method	No
4. HRV-based biofeedback	No

## Conclusion

In our view, there are several mechanisms, responsible for the onset and persistence of COVID-19-related neurological disturbances, which may take place in two phases, in the phase of acute and in the phase of post-acute Sars-CoV-2 infection. We also proposed the point of view by approaching post-COVID-19 complications from the perspective of dynamical system theory. Last but not least, we propose that neurofeedback may represent a potential therapy for post-COVID-19 neurological complications. We have discussed potential limiting factors specific to BFB treatment. Apart from them, we feel it is necessary to mention some general limitation of our considerations. Firstly, there is limited knowledge about overall duration of post-COVID-19 complications and their dynamics. Secondly, it is not clear whether and how the post-COVID-19 complications caused by new variants of SARS-CoV-2 differ in their severity, types and responsiveness to the treatment. Also, the type of particular post-COVID-19 complication might also influence its responsiveness to the treatment. For instance, post-COVID-19 complications caused by direct viral invasion might respond differently to the therapy than post-COVID-19 complications caused indirectly by SARS-CoV-2 effects on CNS. Regarding neurofeedback as proposed possible therapy for neurological post-COVID-19 problems, the exact etiology of these neurological problems may play a crucial role in the level of effectiveness of neurofeedback. It might be of a considerable relevance whether particular neurological problems originate from COVID-19-related damage to brain or whether the particular neurological consequence is the result of extra-neural etiology. Last but not least, it is necessary to say that interdisciplinary approach and combination of other therapies than neurofeedback, for instance rehabilitation, breathing exercise and psychotherapy, should be included for increasing the efficacy of the treatment of post-COVID-19 complications.

All in all, in spite of these limitations, we believe our considerations are worth-studying and we hope they will help to broaden the research horizons in the related neuroscience research.

## Author Contributions

MO wrote the preliminary and final manuscript. EK helped with administration issues. Both authors contributed to the article and approved the submitted version.

## Conflict of Interest

The authors declare that the research was conducted in the absence of any commercial or financial relationships that could be construed as a potential conflict of interest.

## Publisher’s Note

All claims expressed in this article are solely those of the authors and do not necessarily represent those of their affiliated organizations, or those of the publisher, the editors and the reviewers. Any product that may be evaluated in this article, or claim that may be made by its manufacturer, is not guaranteed or endorsed by the publisher.
